# Identification of Novel Diagnosis Biomarkers for Therapy-Related Neuroendocrine Prostate Cancer

**DOI:** 10.3389/pore.2021.1609968

**Published:** 2021-09-27

**Authors:** Cuijian Zhang, Jinqin Qian, Yucai Wu, Zhenpeng Zhu, Wei Yu, Yanqing Gong, Xuesong Li, Zhisong He, Liqun Zhou

**Affiliations:** Department of Urology, Peking University First Hospital Institute of Urology, National Urological Cancer Center, Peking University, Beijing, China

**Keywords:** biomarker, WGCNA, signature, neuroendocrine prostate cancer (NEPC), LASSO, mCRPC

## Abstract

**Background:** Therapy-related neuroendocrine prostate cancer (NEPC) is a lethal castration-resistant prostate cancer (CRPC) subtype that, at present, lacks well-characterized molecular biomarkers. The clinical diagnosis of this disease is dependent on biopsy and histological assessment: methods that are experience-based and easily misdiagnosed due to tumor heterogeneity. The development of robust diagnostic tools for NEPC may assist clinicians in making medical decisions on the choice of continuing anti-androgen receptor therapy or switching to platinum-based chemotherapy.

**Methods:** Gene expression profiles and clinical characteristics data of 208 samples of metastatic CRPC, including castration-resistant prostate adenocarcinoma (CRPC-adeno) and castration-resistant neuroendocrine prostate adenocarcinoma (CRPC-NE), were obtained from the prad_su2c_2019 dataset. Weighted Gene Co-expression Network Analysis (WGCNA) was subsequently used to construct a free-scale gene co-expression network to study the interrelationship between the potential modules and clinical features of metastatic prostate adenocarcinoma and to identify hub genes in the modules. Furthermore, the least absolute shrinkage and selection operator (LASSO) regression analysis was used to build a model to predict the clinical characteristics of CRPC-NE. The findings were then verified in the nepc_wcm_2016 dataset.

**Results:** A total of 51 co-expression modules were successfully constructed using WGCNA, of which three co-expression modules were found to be significantly associated with the neuroendocrine features and the NEPC score. In total, four novel genes, including NPTX1, PCSK1, ASXL3, and TRIM9, were all significantly upregulated in NEPC compared with the adenocarcinoma samples, and these genes were all associated with the neuroactive ligand receptor interaction pathway. Next, the expression levels of these four genes were used to construct an NEPC diagnosis model, which was successfully able to distinguish CRPC-NE from CRPC-adeno samples in both the training and the validation cohorts. Moreover, the values of the area under the receiver operating characteristic (AUC) were 0.995 and 0.833 for the training and validation cohorts, respectively.

**Conclusion:** The present study identified four specific novel biomarkers for therapy-related NEPC, and these biomarkers may serve as an effective tool for the diagnosis of NEPC, thereby meriting further study.

## Introduction

Prostate cancer (PCa) is one of the most prevalent cancers for men in western countries and throughout the world with an estimated incidence of 1.276 million cases in 2018. Owing to the aging population globally, the incidence rate of PCa is expected to increase to 2.3 million by the year 2040, thereby posing an even greater threat to human health [1, 2]. Although the 5 years survival rate of localized PCa is >95%, the overall survival time for the advanced stage of this disease, metastatic castration-resistant prostate cancer (mCRPC), is only 9–36 months [3]. Moreover, nearly 15–20% of mCRPC patients apparently undergo a histological change from the adenocarcinoma to the neuroendocrine subtype following multiple treatments, which is a terminally aggressive PCa subtype [4]. The emergence of neuroendocrine prostate cancer (NEPC) may result in resistance to the androgen receptor (AR)-based therapies, a development that is significantly unfavorable towards patients’ survival [5]. Meanwhile, NEPC patients may develop additional sensitivity to platinum-based chemotherapy, which is the recommended therapeutic intervention to treat NEPC according to the National Comprehensive Cancer Network (NCCN) guidelines [6]. The timely diagnosis of NEPC prevents patients from being subjected to ineffective standard treatments of CRPC, including novel hormone and Taxane-based therapies, and also enables the progression of the disease to be effectively indicated, even in the absence of (or with an underestimated elevation of) prostate-specific antigen (PSA).

At present, the diagnosis of treatment-emergent NEPC is dependent on the histological assessment of tissue biopsies according to the World Health Organization (WHO) classification system of 2004, and neuroendocrine markers, including chromogranin A, synaptophysin (SYP), neuron-specific enolase (NSE), and CD56, are widely used. However, these diagnoses are not only experience-based but also may lead to misdiagnosis due to the presence of mixed tumors (with both adenocarcinoma and NEPC morphologies) or intratumor heterogeneity [7]. Currently, there is no robust molecular diagnosis tool available for NEPC due to its relative rarity and the limited availability of tissue samples. It is worth mentioning that a recognized diagnostic tool that addresses the NEPC scores was used to calculate the scores of a set of 70 genes, and the correlations among their alterations in DNA, RNA, and/or epigenomic status with the NEPC feature [8, 9]. However, though this tool can accurately distinguish NEPC from CRPC, its comprehensive but complex profiling methods are likely to hinder its applicability to a wider range of clinical practices. The features of NEPC include indifference towards the AR signaling pathway, downregulation of known androgen-regulated genes, and overexpression of neuroendocrine-associated genes [10, 11]. For example, co-upregulation of the *LIN28B*, *SOX2*, *EZH2*, and *SPINK1* genes was reported in NEPC tumors [12–14]. Based on the biological findings, further studies, however, are required to combine molecular features for predicting NEPC transformation and to identify patients at a higher risk of developing lineage plasticity. Regrettably, certain of the markers that are in the process of being developed, including neuronal markers such as SYP, enolase 2 (ENO2), chromogranin-A (CHGA), and CD56 [15]; DNA methylation [16, 17]; the alterations in the expression of mRNA [12–14]; and non-coding RNAs [7] exhibit either low accuracy or are at too preliminary a stage for clinical application at the present time. Therefore, the identification of novel and robust biomarkers for the diagnosis of NEPC is urgently required.

**FIGURE 1:**
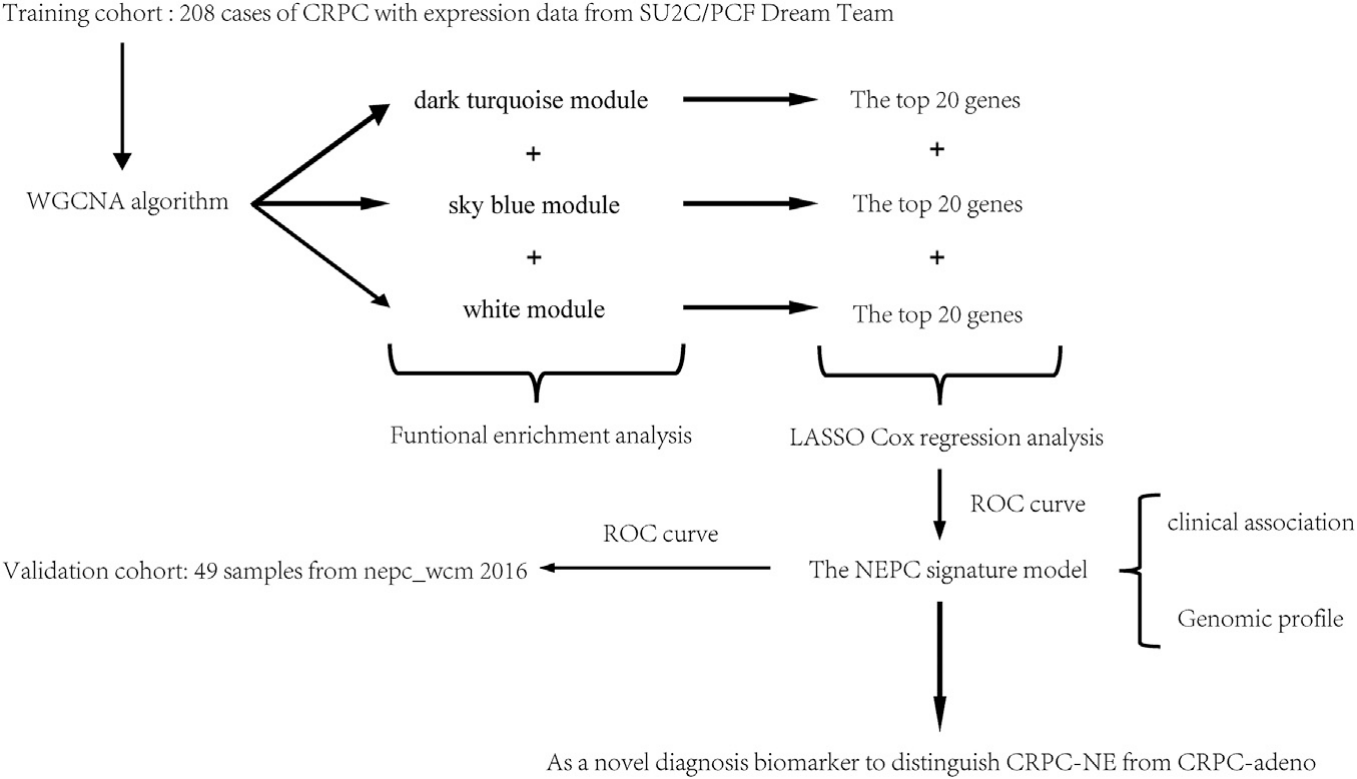
A workflow chart for constructing an NEPC signature model.

In order to establish an effective diagnostic tool for NEPC, in the present study, Weighted Gene Co-expression Network Analysis (WGCNA) and the least absolute shrinkage and selection operator (LASSO) Cox regression analysis were performed to identify correlated gene modules and hub genes in selected modules to construct an NEPC feature-related model, which was subsequently validated in another independent cohort. A flow chart indicating a schematic representation for the approach in this study was shown in Figure 1.

## Methods

### Data Collection

The mRNA expression data and corresponding clinical feature of castration-resistant prostate cancer (CRPC), including castration-resistant prostate adenocarcinoma (CRPC-adeno) and castration-resistant prostate adenocarcinoma with neuroendocrine features (CRPC-NE), were all downloaded from the cBioPortal database (https://www.cbioportal.org/). Specifically, a total of 208 CRPC samples with expression data were gained from SU2C/PCF Dream Team [9] to set as the training dataset, and 49 samples were obtained from the Neuroendocrine Prostate Cancer program (nepc_wcm 2016) [8] and applied as the validation dataset. The clinical features of the training and validation dataset were demonstrated in Table 1.

**TABLE 1:** The clinical information of CRPC samples.

**Variable**		SU2C/PCF Dream team	nepc_wcm 2016
Total		208	49
Age	Median (range)	60.8 [38.6, 80.6]	NA
Mutation count	Median (range)	65 [1, 1,065]	38 [15, 414]
AR score	Median (range)	0.481 [-0.265, 0.694]	NA
Genomic burden	Median (range)	NA	0.34 [0.01, 0.94]
Gleason score	6	13	NA
	7	46	NA
	8	23	NA
	9	65	NA
	10	13	NA
	11	1	NA

CRPC, castration-resistant prostate cancer, NA, not applicable.

### Co-Expression Network Construction using WGCNA and Target Prediction

The WGCNA algorithm was run in the R software package (http://www.r-project.org/) to assess the clinical characteristics of NEPC and their associated modules by calculating the correlation coefficient simultaneously with the minimum gene number of 30 in each module. A power value of 3 was used in this analysis as the package suggested, and values above a pre-determined threshold are considered similar. Each module can be associated with a characteristic according to the eigenvector of the module and the correlation coefficient of the phenotype or the saliency *p*-value of the module. With the application of a network heat map, the connections between the characteristic association module and other modules can be visualized. A hierarchical clustering tree was constructed based on the weighted correlation coefficients of genes and genes with similar patterns in one module.

### Functional Enrichment Analysis of Co-Expression Modules

We uploaded the data to the database for Annotation, Visualization, and Integrated Discovery (DAVID) (https://david.ncifcrf.gov/) for analyzing the function of genes in key co-expression modules which is a classic gene enrichment analysis website, mainly used for differential gene function and pathway enrichment analysis.

### Identification of Hub Genes in Selected Modules

After screening the key gene modules associated with the characteristics of NEPC, the gene co-expression network map was drawn based on the relations of the genes within the modules. For each module, we selected the top 20 genes to identify the hub genes.

### Model Building and Validation

LASSO, which is suitable for the regression of high-dimensional data, was used to select the most useful predictive features from the primary data set [18]. Tuning parameter (L) selection in the LASSO model used 10-fold cross-validation via minimum criteria. The NEPC signature score was calculated by the following formula: NEPC signature score = gene 1 expression × γ_1_ + gene 2 expression × γ_2_ + gene 3 expression × γ_3_ + . + gene n expression × γ_n_, where γ_n_ denotes the coefficient for each hub gene in the multivariate Cox regression model. The area under the receiver operating characteristic curve was plotted using the “timeROC” package in R studio. A coefficient profile plot was produced against the log(L) sequence. A score was calculated for each patient *via* a linear combination of selected genes that were weighted by their respective coefficients.

### Gene Set Enrichment Analysis

The samples from the discovery dataset were divided into high- and low-expression groups according to the genes’ mRNA level, and the median expression level served as a cut-off value. GSEA was performed to identify the molecular feature of the founding biomarker genes [19].

### Statistical Analysis

Statistical analysis was conducted with R software (v. 3.4.3, http://www.Rproject.org). Categorical variables were analyzed by use of the Fisher’s exact test. Continuous variables were analyzed using Student’s *t*-test for paired samples. The median value was used in this work as a cutoff to classify the patients the training and validation cohorts into high- and low-level groups. A *p*-value below 0.05 was considered statistically significant.

## Results

### Weighted Co-Expression Network Construction

**FIGURE 2:**
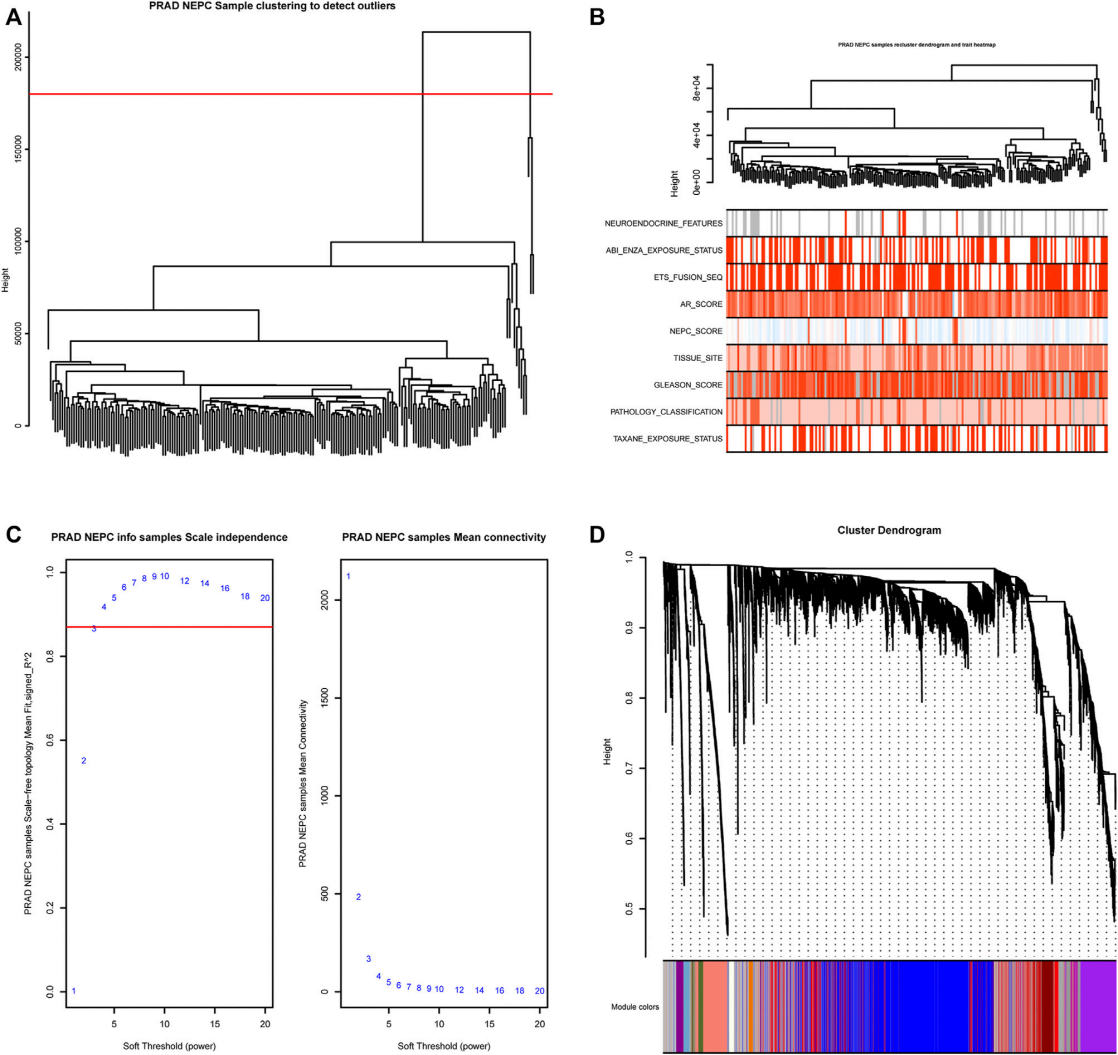
WGCNA module clustering and soft threshold power identification. **(A)** Hierarchical clustering dendrogram of 208 samples based on the Euclidean distance; all samples were included in the analysis. **(B)** The clustering dendrogram and heat map shows the Euclidean distance and sample characteristic correlations. Light color represents a lower value, dark color represents a higher value, and grey represents a missing value. **(C)** Soft threshold power screening based on the network topology, the analysis used a power of 3 as the package suggested, the left panel is the coordinate map of the soft-thresholding power (*x*-axis) and the scale-free fit index (*y*-axis), and the right panel is the coordinate map of the mean connectivity (*y*-axis) and the soft-thresholding power (*x*-axis). **(D)** Co-expression Module clustering and identification dendrogram, 51 modules were identified and each row includes the highly correlated genes of one module.

The gene expression matrix and clinical information of metastatic prostate adenocarcinoma samples were obtained from the SU2C-Prostate Cancer Foundation (PCF) Dream Team (Precision Therapy for Advanced Prostate Cancer). A hierarchical clustering dendrogram of 208 samples was constructed based on the Euclidean distance, all samples were included in the analysis (Figure 2A). Additionally, basic patient information, including the neuroendocrine features, the NEPC score, AR score, Gleason score, tissue site, and exposure status, amongst others, were attached below the resulting tree (Figure 2B). As recommended by the package, the soft threshold power value 3 was used to construct the gene co-expression network (Figure 2C). In total, 51 modules were identified based on co-expression module clustering and the construction of the hierarchical clustering dendrogram (Figure 2D).

## Association of Modules With Clinical Traits

**FIGURE 3:**
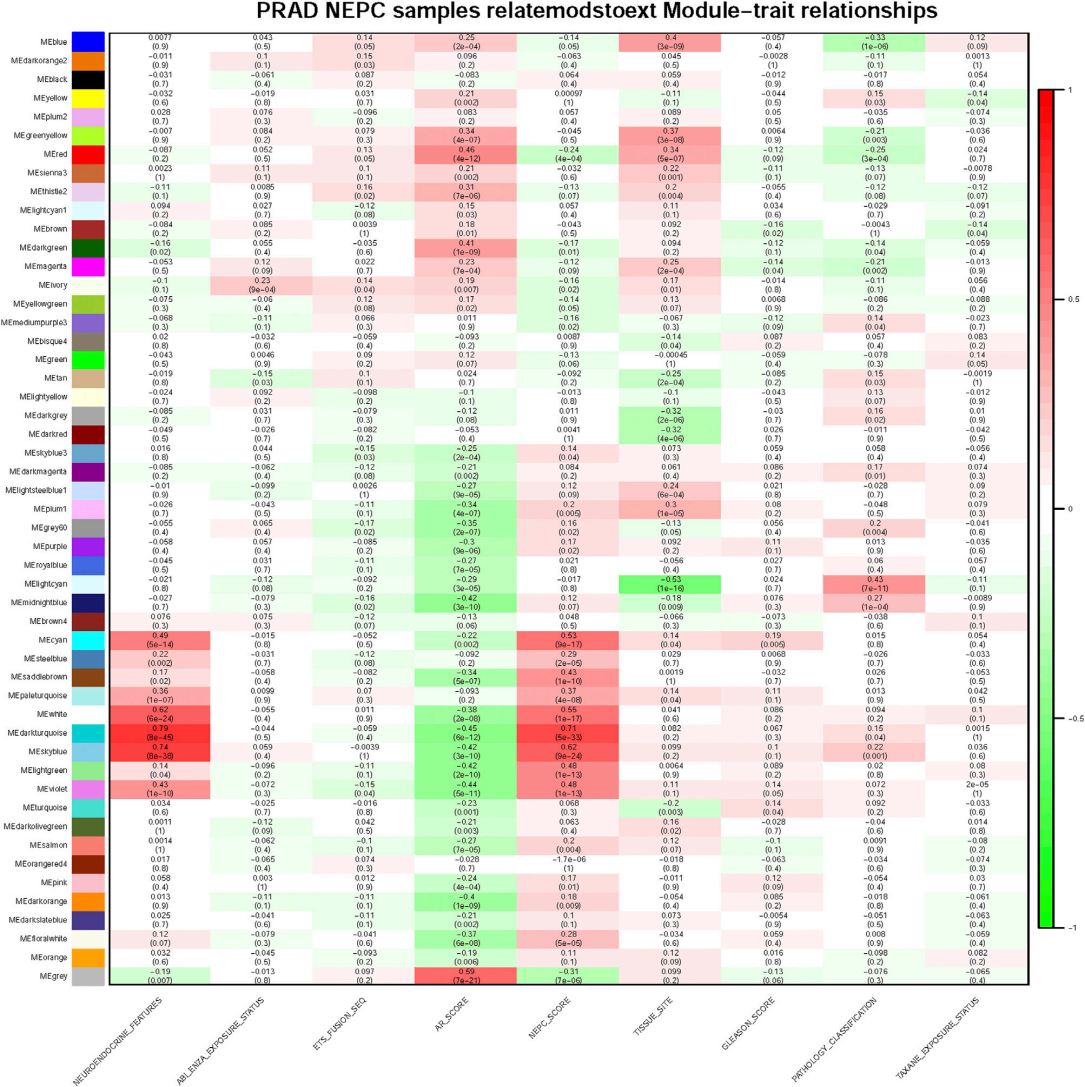
Module and clinical characteristic relationship based on WGCNA. Module and clinical characteristic relationship heat map. In each cell, the correlation between the corresponding module and characteristic are displayed with correlation numbers and colors (red as 0.0 ∼ 1.0, green as −1.0 ∼ 0.0) and *p*-values are also shown in each cell, darker color means higher correlation.

For each module, gene co-expression was summarized according to the eigengene. The correlations of each eigengene were calculated with clinical characteristics, such as Abiraterone and Enzalutamide (ABI ENZA) exposure status, AR score, tissue site, Gleason score, pathology classification, and in particular, the neuroendocrine features and NEPC score. The three highest correlated modules with the neuroendocrine feature were chosen, as denoted by the dark turquoise row (r = 0.71, *p* < 0.05), sky blue row (r = 0.62, *p* < 0.05), and white row (r = 0.55, *p* < 0.05) (Figure 3). Interestingly, each one of these three selected modules was also highly correlated with the NEPC score.

## Enrichment Analysis of the Biological Features

**FIGURE 4:**
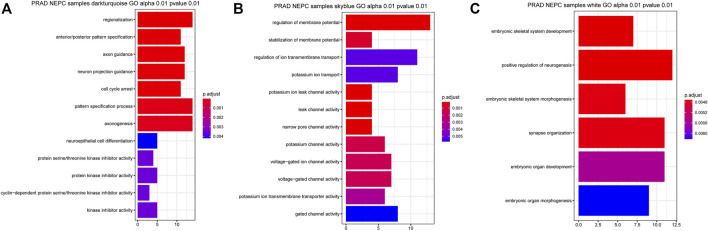
Related signaling pathway of the three most related modules with NEPC score. **(A)** GO analysis of the dark turquoise module **(A)**, sky blue module **(B)**, and white module **(C)**. The color indicates the significant degree of enrichment and the size indicates the number of genes enriched for each result.

Gene Ontology (GO) analysis of genes in the three selected identified modules was performed, clarifying the combined features that were associated with biological processes (BP), molecular functions (MF), and cellular components (CC). The genes in the dark turquoise module were mainly associated with “regionalization”, “anterior/posterior pattern specification”, “axon guidance”, “neuron projection guidance”, “cell cycle arrest”, and “pattern specification process and axon genesis” (Figure 4A). By contrast, the genes in the sky-blue module were mainly enriched in the regulation of “membrane potential”, “stabilization of membrane potential”, “potassium ion leak channel activity”, “leak channel activity”, “narrow pore channel activity”, “potassium channel activity”, “voltage-gated ion channel activity”, and “voltage-gated channel activity” (Figure 4B). Finally, the genes in the white module were involved in “embryonic skeletal system development”, “positive regulation of neurogenesis”, “embryonic skeletal system morphogenesis”, and “synapse organization” (Figure 4C).

## Identification and Validation of Hub Genes

**FIGURE 5:**
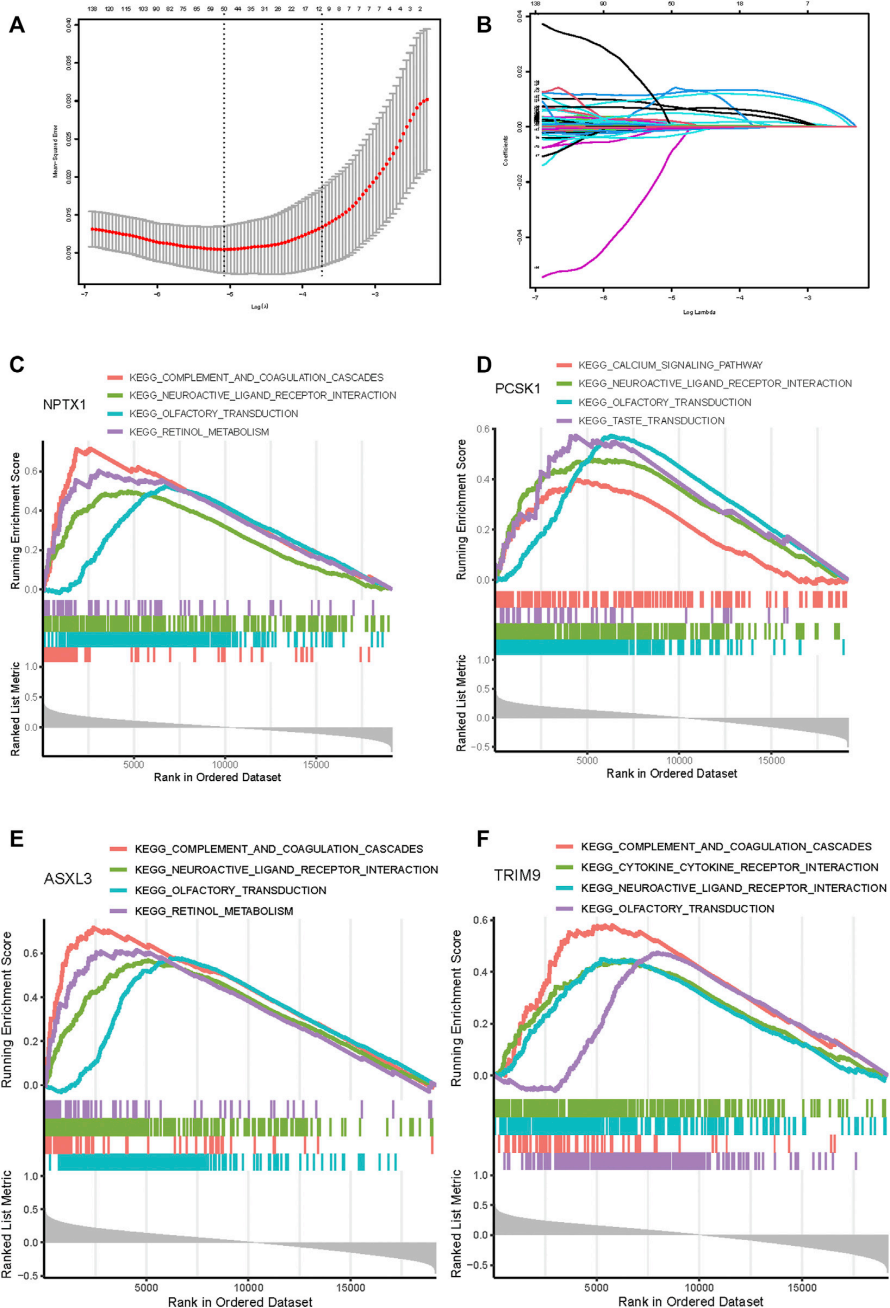
Feature genes selection using the least absolute shrinkage and selection operator (LASSO) binary logistic regression model. **(A)** The result of LASSO regression analysis. **(B)** LASSO coefficient profiles of the 60 genes. A coefficient profile plot was produced against the log(L) sequence. Gene set enrichment analysis for NPTX1 **(C)**, PCSK1 **(D)**, ASXL3 **(E)**, and TRIM9 **(F)**. The top four pathways enriched in the high expression group are shown.

Subsequently, 314 intra-module connectivity genes were screened as candidate genes from the dark turquoise, sky-blue, and white modules. The top 60 genes were selected (Supplementary Table S1) for LASSO regression analysis, comprising the top 20 hub genes from each of the three selected modules. In total, four potential hub genes, including neuronal pentraxin 1 (*NPTX1*), proprotein convertase subtilisin/kexin type 1 (*PCSK1*), ASXL transcriptional regulator 3 (*ASXL3*), and tripartite motif-containing 9 (*TRIM9*) were identified as non-zero coefficients in the LASSO logistic regression model used to predict NEPC feature (Figures 5A, B). As shown in Figures 5C–F, these four identified genes were all associated with the neuroactive ligand-receptor interaction and olfactory transduction pathways. With the exception of *PCSK1,* the other three genes (*NPTX1*, *ASXL3, and TRIM9*) were all associated with complement and coagulation cascades. Additionally, *PCSK1* was also associated with the calcium signaling pathway.

**FIGURE 6:**
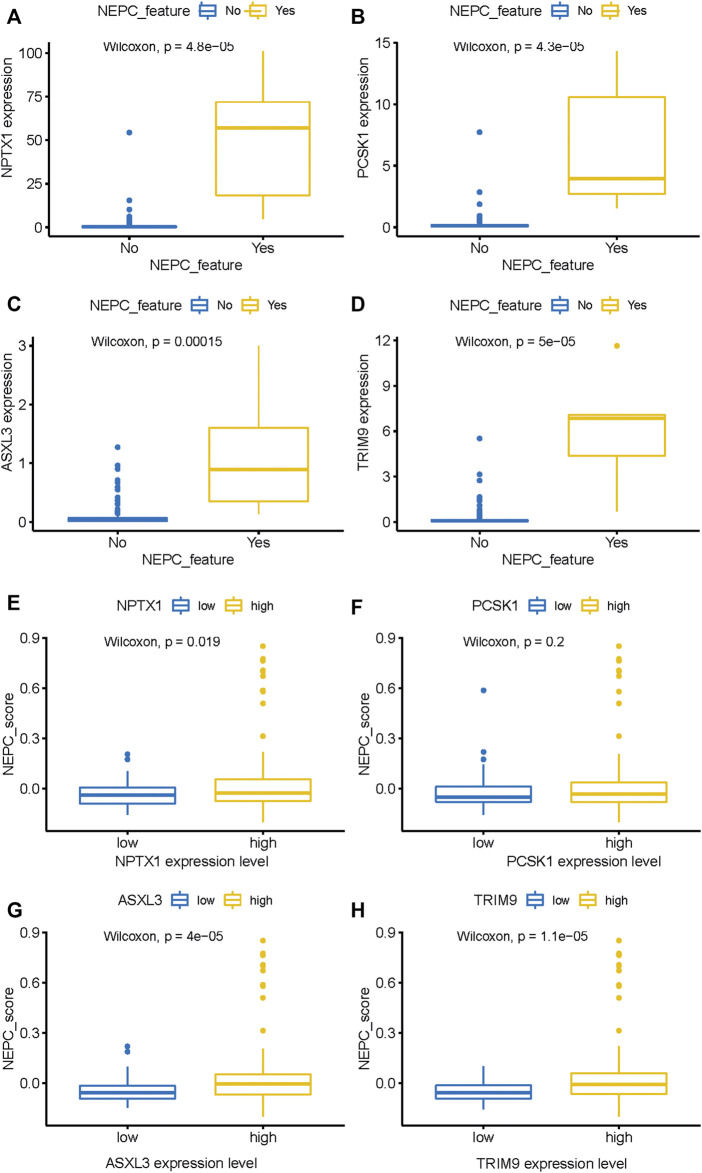
The expression level of four genes between samples with NEPC feature or not from the SU2C dataset. NPTX1 **(A)**, PCSK1 **(B)**, ASXL3 **(C)**, and TRIM9 **(D)** expression level in samples with (N = 6) or without (N = 161) NEPC feature. The relationship between the expression level of identified biomarkers [NPTX1 **(E)**, PCSK1 **(F)**, ASXL3 **(G)**, and TRIM9 **(H)**] and the NEPC score. The median value of gene expression level was used as a cutoff to classify samples into high- (N = 104) and low- (N = 104) expression level groups.

Through the analysis of the NEPC features in conjunction with the expression levels of the hub genes, it was demonstrated that *NPTX1*, *PCSK1*, *ASXL3,* and *TRIM9* were all significantly upregulated in samples with NEPC feature (*p* < 0.001, Figures 6A–D). With the exception of *PCSK1*, the expressions of the other three genes (*NPTX1*, *ASXL3,* and *TRIM9*) were all positively correlated with the traditional NEPC score (Figures 6E–H). Based on the aforementioned results, the NEPC signature was established by the expression of these four hub genes, consisting of *NPTX1*, *PCSK1*, *ASXL3,* and *TRIM9* (Table 2).

**TABLE 2:** The coefficients of identified genes in the NEPC signature.

Gene	Coefficient	*p*-value
NPTX1	0.003247	0.028
PCSK1	0.003142	0.011
ASXL3	0.024173	0.028
TRIM9	0.020398	0.011

**FIGURE 7:**
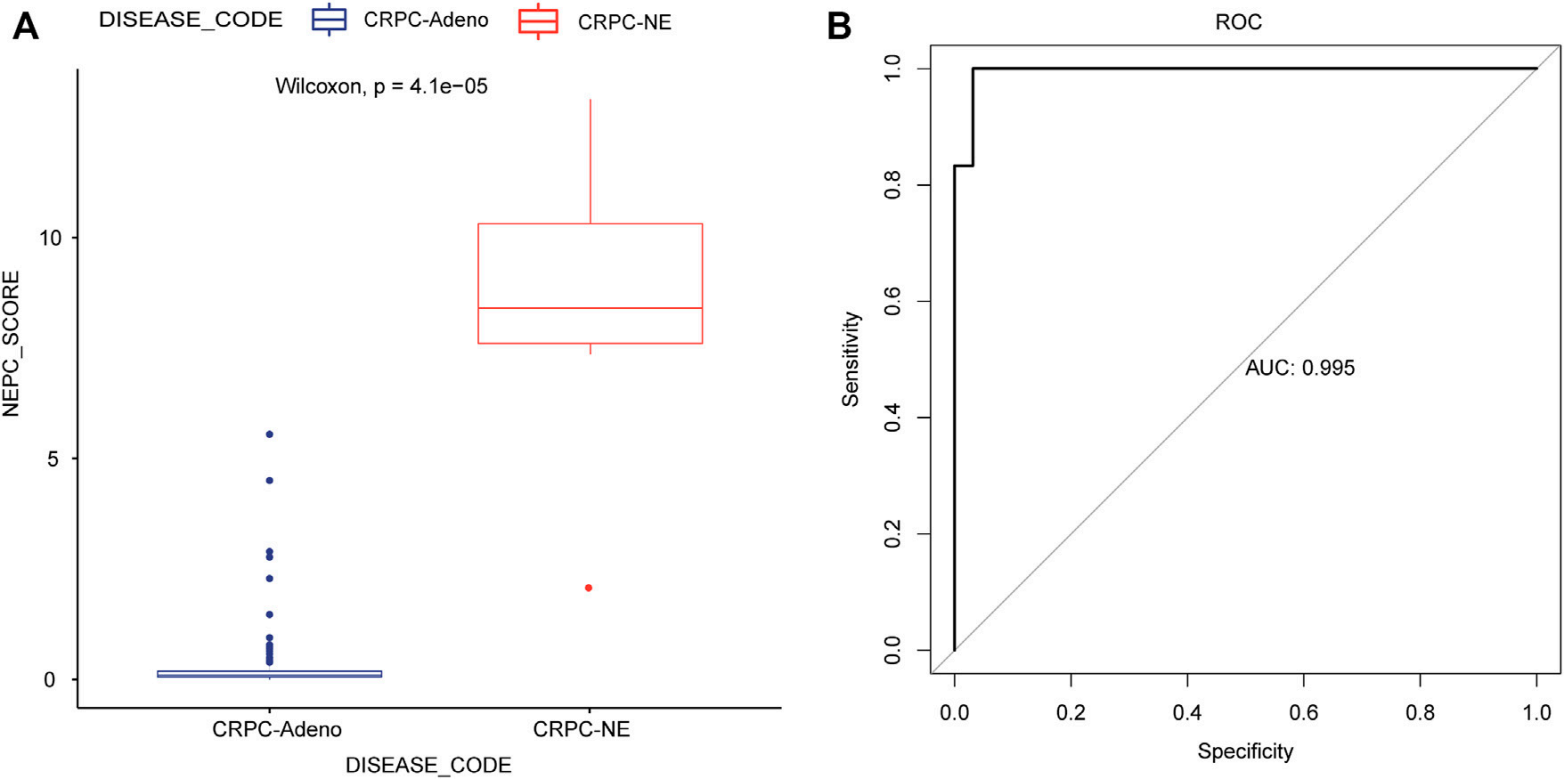
Application and evaluation of the NEPC predicted signature model. **(A)** The differences in predicted signature score between CRPC-NE and CRPC-adeno samples. **(B)** The ROC curves for NEPC predicted signature model in the training cohort.

In addition, the predicted signature model was applied in the training dataset, and this analysis demonstrated that the CRPC-NE samples had a significantly higher predicted signature score, compared with the CRPC-adeno samples (Figure 7A). The AUC of the NEPC prediction was 0.995, showing its strong plausibility as the NEPC diagnosis signature (Figure 7B).

### Validation of the Established NEPC Signature

**FIGURE 8:**
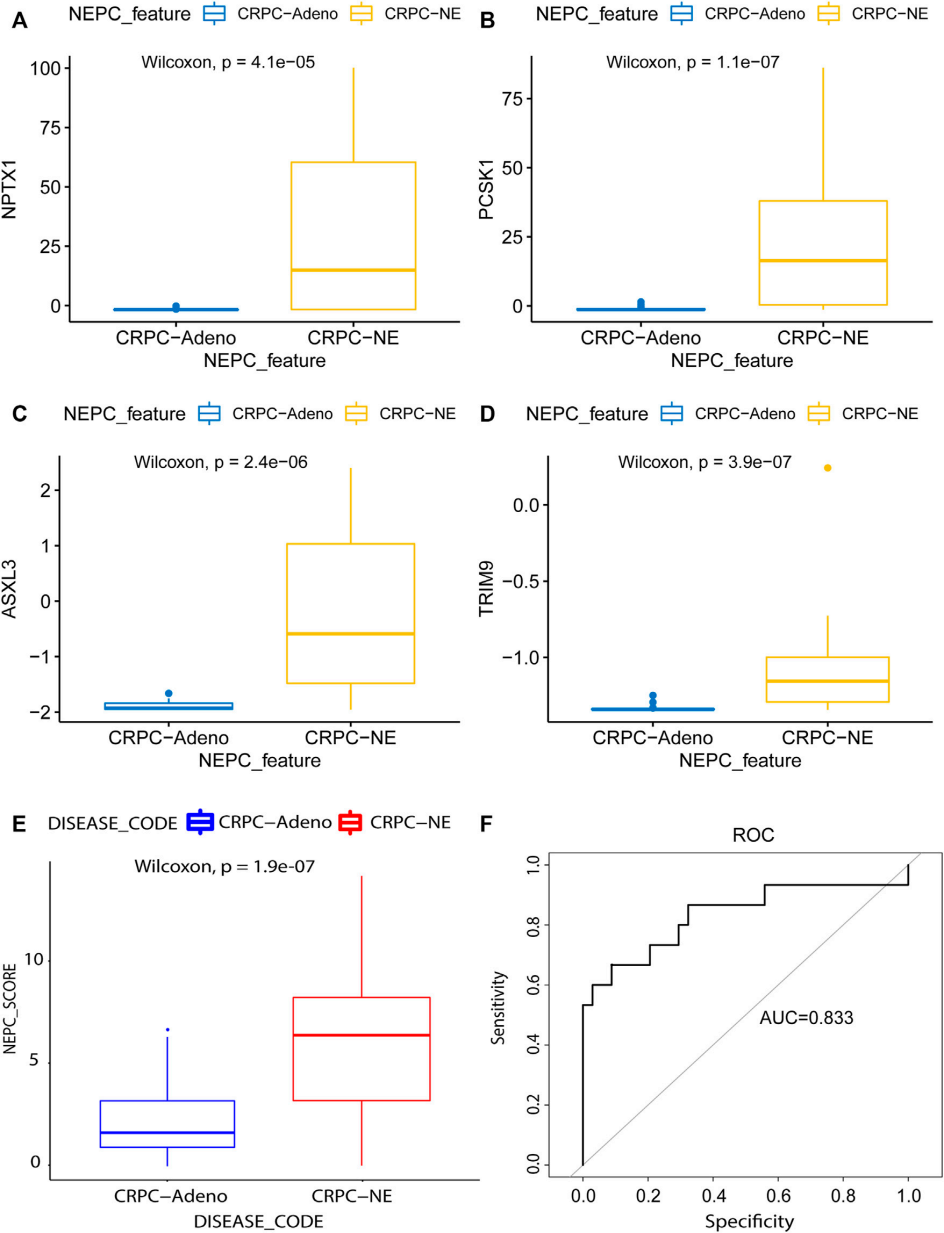
Model validation using the lasso method in an independent dataset. The mRNA differential expression analysis of four potential predicting genes between the samples with (N = 15) or without (N = 34) NE feature in the nepc_wcm_2016 dataset. **(A)** NPTX1. **(B)** PCSK1. **(C)** ASXL3. **(D)** TRIM9. **(E)** The differences in predicted signature score between NEPC and Adenocarcinoma samples. **(F)** NEPC feature-dependent ROC curves were performed in the validation cohort.

Next, in the validation dataset of an independent NEPC program (nepc_wcm_2016), it was noted that patient samples with NEPC features (i.e., the CRPC-NE samples) had significantly higher expression levels of the four genes compared with the CRPC-adeno patient samples (*p* < 0.001, Figures 8A–D). Furthermore, the NEPC samples in the validation cohort (nepc_wcm 2016) also had significantly higher predicted signature scores compare with those in the CRPC-adeno samples (Figure 8E). Meanwhile, the AUC of the NEPC prediction was 0.833, which confirmed the validity of this model in terms of its accuracy for the diagnosis of NEPC (Figure 8F).

## Genomic Differences Between Samples With High and Low NEPC Signature Scores

**FIGURE 9:**
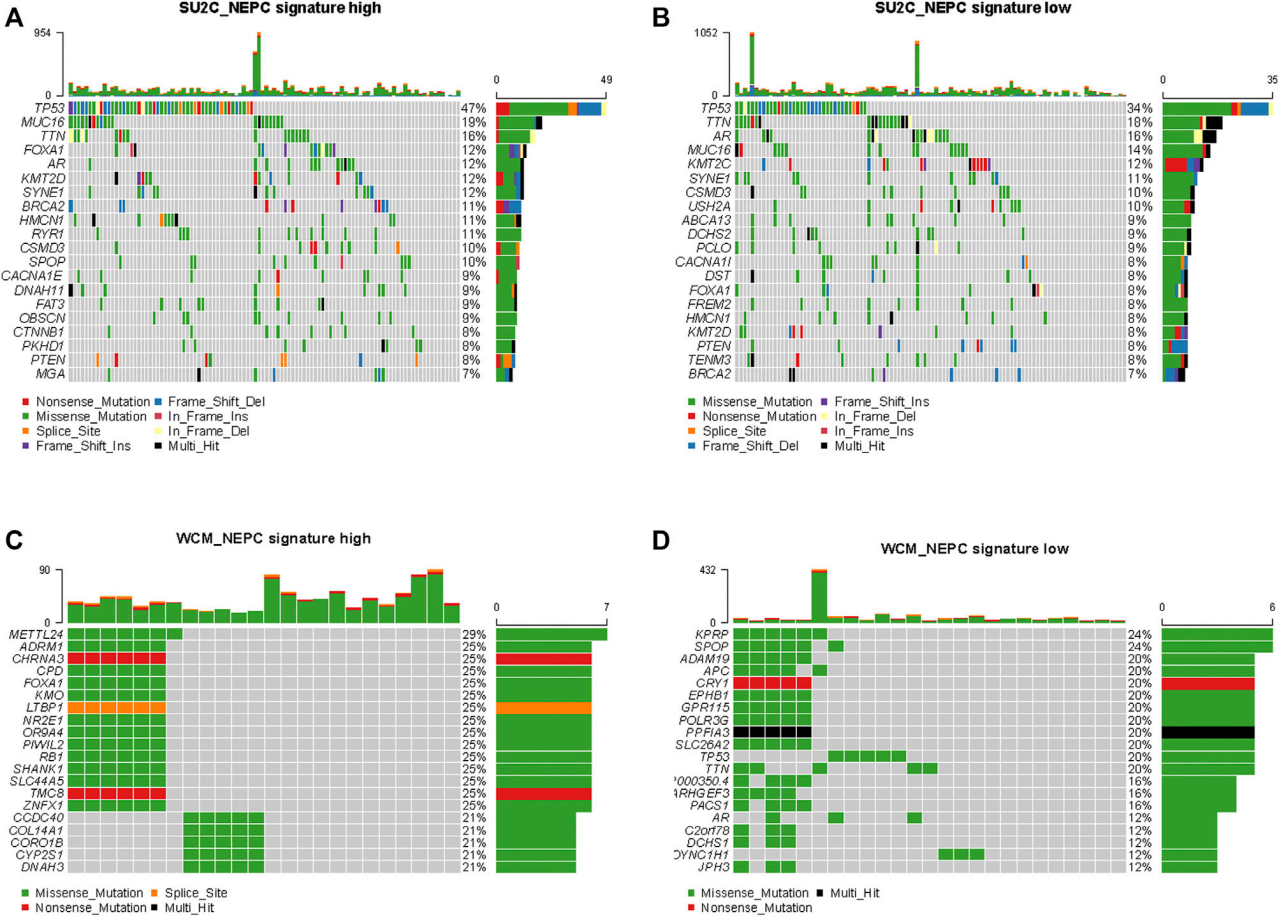
Genomic alterations in samples with high and low NEPC signature. The oncoprint results of samples with high **(A)** and low **(B)** NEPC signature of SU2C dataset. The oncoprint results of samples with high **(C)** and low **(D)** NEPC signature of WCM dataset. The top 20 most prevalent genes were presented.

The top 20 mutated genes in samples with high and low NEPC signature scores from the SU2C-PCF dataset were shown in Figures 9A, B. No significant differences were identified in the prevalence of altered genes comparing between these two groups, including genes associated with the AR signaling pathway (*AR* and *FOXA1*). However, in the validation dataset, a significantly higher prevalence of certain genes, including *RB1*, *METTL24*, and *ADRM1*, were identified in the samples with a high NEPC signature score (Figure 9C). On the other hand, *KPRP* and *SPOP* were identified as more prevalent genes in the CRPC-adeno samples (Figure 9D).

## Discussion

NEPC is a highly aggressive subtype of prostate cancer that is associated with poor survival and developing resistance to novel hormone treatments, including the administration of abiraterone and enzalutamide. Early diagnosis of NEPC and the timely application of treatment for advanced NEPC are difficult to accomplish, however, due to a deficiency of robust molecular biomarkers [20]. The previously established NEPC score, which was comprehensively analyzed on the basis of DNA alteration, methylation, and RNA expression parameters, has been shown to enable the precise identification of CRPC-NE [8, 9]. Although this model was effective, however, it was not appropriate for clinical popularization or application due to its costliness and its complex algorithm. In order to find a concise but effective diagnosis model for NEPC, the present study aimed to analyze the RNA-seq data of therapy-related NEPC compared with CRPC.

Biological analysis of our identified gene modules has suggested that neurite extension, as the neuroendocrine marker, is a highly correlated pathway in the modules. Changes in membrane potential and ion channel activity may occur during the process of NE differentiation in which the overexpression of alpha (1H) mRNA (i.e., a single type of LVA calcium channel mRNA) was reported during neuroendocrine differentiation [21]. Furthermore, calcium channels have been shown to facilitate neurite lengthening via promoting basal calcium entry at the resting membrane potential [22-24].

Ultimately, four novel biomarkers for NEPC were identified, including *NPTX1, PCSK1, ASXL3,* and *TRIM9*, by LASSO regression analysis, and it was noteworthy that some of these genes have already been previously identified in the NEPC. Through exon array profiling, Tsai and others identified that 87% (13/16) of NEPC patients had outlier expression of *NPTX1* and *PCSK*1, findings that were also in support of the strong plausibility of these two candidates as markers for the diagnosis of NEPC [25]. *NPTX1* encoded a secreted glycoprotein of size 47–50 kDa and was first identified in the central nervous system as a member of the pentraxin family [26]. Based on mass-spectrometry-based proteomics, it was identified that the level of NPTX1, which is involved in neurogenesis, was elevated in NEPC samples [27]. Recently, an increasing number of studies have shown that *NPTX1* is involved in the progression of various cancers, including lung cancer [28], colon cancer [29], and gastric cancer [30]. *PCSK1*, encoding prohormone convertase 1, belongs to the proprotein convertase family, and its overexpression has been revealed in various subtypes of neuroendocrine tumors [31–33]. Previous studies have shown that treatment-related NEPC also exhibits a high expression of *PCSK1*, and the pattern of promoter methylation was observed to be different among distinct phenotypes of PCa [34, 35]. On the other hand, the role of *ASXL3* and *TRIM9* in the diagnosis of NEPC has not been previously shown. *ASXL3*, the polycomb group (PcG) protein, is essential for neuroendocrine (NE) lung cancer development and was shown via KEGG analysis to be associated with the multiple neuron differentiation signaling pathways [36, 37]. Furthermore, *ASXL3* was found to serve as an effective biomarker for predicting the sensitivity towards BET protein inhibitors in small cell lung cancer (SCLC), highlighting its potential as an actionable biomarker [38, 39]. *TRIM9*, encoding a brain-specific E3 ubiquitin ligase, has been shown by GO analysis to be involved in the neurological disease and inflammation pathways [40]. Its overexpression promotes cell proliferation, and inhibits cell apoptosis via the NF-κB signaling pathway in uterine leiomyoma [41]. As an important transcription factor, NF-κB has also been implicated in the acquisition of neuroendocrine characteristics in prostate cancer cells [42].

Although several diagnosis markers have now been identified, histological assessment with neuroendocrine differentiation remains “the gold standard” for clinical diagnosis of NEPC. However, the clinical and cellular heterogeneity of NEPC patients causes many difficulties for the clinical management of NEPC, and the sampling process of histological assessment is both invasive and painful to patients [43]. Recently, comprehensive genomic profiling revealed molecular heterogeneity in the genomic landscape of metastatic CRPC [8, 9]. The results of the present study should prove to be of great benefit in terms of improving clinical applicability. Furthermore, the novel NEPC signature that has been identified will have the advantage of overcoming the heterogeneity that leads to inaccuracies in the diagnosis of patients with NEPC. Finally, the high AUC value that was determined provides confirmatory evidence that the NEPC signature is trustworthy in terms of its putative role as a novel diagnostic tool to distinguish samples of patients with CRPC-NE from those with CRPC-adeno. However, it should be acknowledged that the present study has some limits because of the sample sizes. Moreover, this study was conducted based on public databases. Therefore, we having been collecting the NEPC patient samples in our hospital and would validate the NEPC signature scoring system as a diagnostic tool in an independent cohort. At the same time, the merits of this NEPC signature scoring system would be assessed with regard to its application in the clinic. To corroborate these findings, the functional roles of the identified four hub genes should be explored in relevant cell lines or in a mouse model. Furthermore, other biomarkers, such as low expression of androgen-regulated genes [e.g., *KLK3* (PSA), *TMPRSS2*, and *NXK3.1*] and high expression of neuroendocrine-associated genes (e.g., *CGA* and *SYP*), may be investigated subsequently to provide further confirmatory evidence for the accuracy of the NEPC signature in the diagnosis of NEPC.

In conclusion, the present study has investigated co-expressed gene modules that were highly correlated with the NEPC score, and four hub genes were screened. The findings obtained have improved our understanding of the underlying molecular mechanism of NEPC. As a model, these hub genes could represent a novel diagnostic marker and therapeutic target for NEPC.

## Data Availability

The datasets presented in this study can be found in online repositories. The names of the repository/repositories and accession number(s) can be found in the article/Supplementary Material.
