# Supplementary material for: Identification of Novel Diagnosis Biomarkers for Therapy-Related Neuroendocrine Prostate Cancer
Source: Pathol Oncol Res. 2021 Sep 27;27:1609968. doi: 10.3389/pore.2021.1609968 (PMC8503838; doi:10.3389/pore.2021.1609968)
Supplement: Supplementary file 1 [file DataSheet1.docx]

**Table S1.** Hub genes identified from the 3 selected modules for LASSO analysis.

| **Module** | **Hub genes** | **Ensemble ID** |
| --- | --- | --- |
| **dark turquoise** | *CACNA1A* | [ENSG00000141837](http://www.ensembl.org/id/ENSG00000141837) |
|  | *BTBD17* | [ENSG00000204347](http://www.ensembl.org/id/ENSG00000204347) |
|  | *CRMP1* | [ENSG00000072832](http://www.ensembl.org/id/ENSG00000072832) |
|  | *ERFE* | [ENSG00000178752](http://www.ensembl.org/id/ENSG00000178752) |
|  | *RASAL1* | [ENSG00000111344](http://www.ensembl.org/id/ENSG00000111344) |
|  | *C1orf127* | [ENSG00000175262](http://www.ensembl.org/id/ENSG00000175262) |
|  | *EFR3B* | [ENSG00000084710](http://www.ensembl.org/id/ENSG00000084710) |
|  | *GRIK3* | [ENSG00000163873](http://www.ensembl.org/id/ENSG00000163873) |
|  | *NKX2.1* | [ENSG00000136352](http://www.ensembl.org/id/ENSG00000136352) |
|  | *INSM1* | [ENSG00000173404](http://www.ensembl.org/id/ENSG00000173404) |
|  | *MYCL* | [ENSG00000116990](http://www.ensembl.org/id/ENSG00000116990) |
|  | *NKAIN2* | [ENSG00000188580](http://www.ensembl.org/id/ENSG00000188580) |
|  | *HOXB8* | [ENSG00000120068](http://www.ensembl.org/id/ENSG00000120068) |
|  | *TAGLN3* | [ENSG00000144834](http://www.ensembl.org/id/ENSG00000144834) |
|  | *HCN4* | [ENSG00000138622](http://www.ensembl.org/id/ENSG00000138622) |
|  | *NPTX1* | [ENSG00000171246](http://www.ensembl.org/id/ENSG00000171246) |
|  | *RPRM* | [ENSG00000177519](http://www.ensembl.org/id/ENSG00000177519) |
|  | *HS3ST6* | [ENSG00000162040](http://www.ensembl.org/id/ENSG00000162040) |
|  | *PCSK1* | [ENSG00000175426](http://www.ensembl.org/id/ENSG00000175426) |
|  | *GRP* | [ENSG00000134443](http://www.ensembl.org/id/ENSG00000134443) |
| **sky blue** | *KCNC1* | [ENSG00000129159](http://www.ensembl.org/id/ENSG00000129159) |
|  | *TRIM9* | [ENSG00000100505](http://www.ensembl.org/id/ENSG00000100505) |
|  | *ZDHHC22* | [ENSG00000177108](http://www.ensembl.org/id/ENSG00000177108) |
|  | *CABP7* | [ENSG00000100314](http://www.ensembl.org/id/ENSG00000100314) |
|  | *NKX2.8* | [ENSG00000136327](http://www.ensembl.org/id/ENSG00000136327) |
|  | *GPC3* | [ENSG00000147257](http://www.ensembl.org/id/ENSG00000147257) |
|  | *NPTX2* | [ENSG00000106236](http://www.ensembl.org/id/ENSG00000106236) |
|  | *ISM2* | [ENSG00000100593](http://www.ensembl.org/id/ENSG00000100593) |
|  | *KCNK10* | [ENSG00000100433](http://www.ensembl.org/id/ENSG00000100433) |
|  | *DRD2* | [ENSG00000149295](http://www.ensembl.org/id/ENSG00000149295) |
|  | *STK32A* | [ENSG00000169302](http://www.ensembl.org/id/ENSG00000169302) |
|  | *ZNF488* | [ENSG00000265763](http://www.ensembl.org/id/ENSG00000265763) |
|  | *ANK2* | [ENSG00000145362](http://www.ensembl.org/id/ENSG00000145362) |
|  | *ILDR2* | [ENSG00000143195](http://www.ensembl.org/id/ENSG00000143195) |
|  | *PNMA3* | [ENSG00000183837](http://www.ensembl.org/id/ENSG00000183837) |
|  | *ATOH7* | [ENSG00000179774](http://www.ensembl.org/id/ENSG00000179774) |
|  | *L1CAM* | [ENSG00000198910](http://www.ensembl.org/id/ENSG00000198910) |
|  | *CLCNKB* | [ENSG00000184908](http://www.ensembl.org/id/ENSG00000184908) |
|  | *ASXL3* | [ENSG00000141431](http://www.ensembl.org/id/ENSG00000141431) |
|  | *ZNF711* | [ENSG00000147180](http://www.ensembl.org/id/ENSG00000147180) |

***Table S2.*** *Continued*

| **Module** | **Hub genes** | **Ensemble ID** |
| --- | --- | --- |
| **white** | *SFTPB* | [ENSG00000168878](http://www.ensembl.org/id/ENSG00000168878) |
|  | *SLC6A20* | [ENSG00000163817](http://www.ensembl.org/id/ENSG00000163817) |
|  | *CALCA* | [ENSG00000110680](http://www.ensembl.org/id/ENSG00000110680) |
|  | *HTR2B* | [ENSG00000135914](http://www.ensembl.org/id/ENSG00000135914) |
|  | *CTTNBP2* | [ENSG00000077063](http://www.ensembl.org/id/ENSG00000077063) |
|  | *ZACN* | [ENSG00000186919](http://www.ensembl.org/id/ENSG00000186919) |
|  | *BMP5* | [ENSG00000112175](http://www.ensembl.org/id/ENSG00000112175) |
|  | *DRD1* | [ENSG00000184845](http://www.ensembl.org/id/ENSG00000184845) |
|  | *HMP19* | [ENSG00000170091](http://www.ensembl.org/id/ENSG00000170091) |
|  | *HRH3* | [ENSG00000101180](http://www.ensembl.org/id/ENSG00000101180) |
|  | *AMPH* | [ENSG00000078053](http://www.ensembl.org/id/ENSG00000078053) |
|  | *ASCL1* | [ENSG00000139352](http://www.ensembl.org/id/ENSG00000139352) |
|  | *MTMR7* | [ENSG00000003987](http://www.ensembl.org/id/ENSG00000003987) |
|  | *IL17C* | [ENSG00000124391](http://www.ensembl.org/id/ENSG00000124391) |
|  | *PITPNM2* | [ENSG00000090975](http://www.ensembl.org/id/ENSG00000090975) |
|  | *SLC4A11* | [ENSG00000088836](http://www.ensembl.org/id/ENSG00000088836) |
|  | *TMEM255B* | [ENSG00000184497](http://www.ensembl.org/id/ENSG00000184497) |
|  | *BAALC* | [ENSG00000164929](http://www.ensembl.org/id/ENSG00000164929) |
|  | *POU3F2* | [ENSG00000184486](http://www.ensembl.org/id/ENSG00000184486) |
|  | *RNF183* | [ENSG00000165188](http://www.ensembl.org/id/ENSG00000165188) |
